# Epidemiology, associated factors and outcomes of ICU-acquired infections caused by Gram-negative bacteria in critically ill patients: an observational, retrospective study

**DOI:** 10.1186/s12871-015-0106-9

**Published:** 2015-09-21

**Authors:** Cosimo Chelazzi, Eleonora Pettini, Gianluca Villa, A. Raffaele De Gaudio

**Affiliations:** Department of Health Sciences, Section of Anaesthesiology and Intensive Care, University of Florence, Florence, Italy

## Abstract

**Background:**

Gram-negative bacteria are increasingly responsible for nosocomial infections, including ICU-acquired infections. Due to high virulence, rate of multi-drug resistance and limited availability of new agents, these infections create cumbersome clinical burdens, making it important to reduce the risk of their occurrence. The aim of the study was to assess epidemiology-related factors and outcomes of Gram-negative, ICU-acquired infections in a cohort of medical-surgical patients.

**Methods:**

A retrospective survey was conducted on all patients admitted to a mixed ICU from January 2012 to December 2013. ‘ICU-acquired infections’ were defined as new infections acquired no less than 48 h after ICU admission. Diagnosis was made according to the Centers for Disease Control and Prevention National Healthcare Safety Network (CDC/NHSN) criteria. Differences across patients who did and did not acquire a Gram-negative infection were tested regarding age, sex, body mass index, medical or surgical admission, cardiovascular comorbidities, chronic obstructive pulmonary disease, diabetes, end-stage renal failure, co-existing tumours and prophylactic anti-fungal treatment. Multivariate analysis was used to assess the independency of these associations. Finally, differences in ICU-mortality, ICU-length of stay and duration of mechanical ventilation were tested across patients with and without new, ICU-acquired, Gram-negative infections.

**Results:**

Of 494 patients admitted to the ICU, 46 (9.3 %) acquired an infection 48 or more hours after admittance. In 30/46 patients (65.2 %) the isolated bacterium was Gram-negative. Univariate analysis showed that clinical factors associated with new ICU-acquired Gram-negative infections were medical admission (*p* < 0.001, 95 % CI 0.59 – 0.29, OR = 0.13), chronic kidney disease (*p* = 0.018, 95 % CI 1.20 – 7.34, OR = 2.98) and prophylactic antifungal therapy (*p* < 0.001, 95 % CI 1.91 – 9.79, OR = 4.33). At multivariate analysis, only medical admission and prophylactic antifungal therapy were significantly associated with ICU-acquired Gram-negative infections. Higher ICU-length of stay and longer duration of mechanical ventilation were associated with these infections while ICU-mortality did not significantly differ.

**Conclusions:**

ICU-acquired Gram-negative infections were common in a cohort of mixed medical-surgical patients. Only medical admission and anti-fungal prophylaxis were found to be independently associated with these infections; they were not found to have a significant effect on ICU-mortality.

## Background

Prevalence of infections in ICU patients worldwide is an estimated 51.4 % [1]. Gram-negative bacteria are largely responsible for ICU-acquired infections and extended- (XDR) or multi-drug resistant (MDR) strains are increasingly the ones isolated, including carbapenemase-producing *Klebsiella pneumoniae* (KPC), *Acinetobacter spp.* and *Pseudomonas aeruginosa* [2, 3]. Infections due to XDR/MDR bacteria lead to higher ICU-mortality rates, increased morbidity and increased healthcare costs, while only limited therapeutic options are available [4–6]. There are many reported risk factors for ICU-acquired infections. These may include patient-related comorbidities such as diabetes mellitus or chronic respiratory conditions, and nosocomial factors such as empiric use of fluoroquinolones, immunosuppression and the use of invasive devices [7–9]. Interestingly, bacterial and fungal floras may interact in the same patient, competing with each other and reciprocally controlling each other’s growth [10]. *Candida albicans* derived farnesol may inhibit the growth of *A. baumannii, E. coli, P. aeruginosa* and *S. aureus* [10]. Prophylactic use of antifungal drugs may alter the bacterial-fungal balance and potentially contribute to bacterial infections.

The aim of this study was to determine the epidemiology of Gram-negative, ICU-acquired infections, to assess the factors to acquisition, including prophylactic use of antifungals, and to assess the effect of these infections on ICU mortality, ICU-length of stay and days of mechanical ventilation.

## Methods

### Study design and setting

A monocentric, observational, retrospective study was conducted for a cohort of patients admitted to a six-bed mixed medical-surgical ICU of a tertiary referral hospital between January 2012 and December 2013. The institutional Ethical Board of Careggi University Hospital (Florence, Italy) reviewed and approved this protocol (n° 2013/0024940) and waived the need for informed consent due to the retrospective nature of the study. The study was designed following the indications of STROBE guidelines.

### Patients and definitions

Patients whose ICU-length of stay was longer than 48 h were considered eligible for our study. ‘ICU-acquired infections’ were defined as those acquired no less than 48 h after ICU admission. Clinical diagnosis was made according to the criteria of the Centers for Disease Control and Prevention National Healthcare Safety Network (CDC/NHSN) [11]. All microbiological isolates of infected patients were evaluated. In cases where a patient acquired more than one infection, the first diagnosed infection was defined as ‘primary’ and the subsequent infections as ‘secondary’. ICU patients admitted for more than 48 h without evidence of infection or those who acquired infections prior to 48 h from admittance were treated as ‘controls’ in the subsequent analysis.

### Objectives

The first objective of this study was to assess the epidemiology of Gram-negative, ICU-acquired infections. Epidemiology was described in terms of incidence of new infections, aetiology, and site distribution.

The second objective was to assess which clinical factors were associated with these infections. Differences between patients who did and did not acquire a Gram-negative infection were tested according to a number of demographic and clinical factors, including: age, sex, body mass index, type of admission (medical/surgical), cardiovascular comorbidities, chronic respiratory impairment, diabetes mellitus, end-stage renal failure, co-existing tumours (metastatic/non-metastatic), infection/sepsis at ICU admittance and anti-fungal prophylaxis administered prior to the Gram-negative infection. ‘Surgical’ patients were defined as those who had surgery in the 24 h prior to ICU admittance. All other patients were defined as ‘medical’. Cardiovascular comorbidities included hypertension, chronic heart failure, valvular disease and coronary artery disease. ‘Chronic respiratory impairment’ was defined as chronic respiratory failure inducing symptoms. ‘End-stage renal failure’ was defined as the chronic need for dialysis. ‘Anti-fungal prophylaxis’ was defined as previous administration of anti-fungal agents in the absence of microbiological or serological evidence of fungal infection [12].

The third objective was to assess the outcomes of patients with ICU-acquired Gram-negative infections. ICU mortality, ICU-length of stay and duration of mechanical ventilation were considered, and differences across patients were tested.

### Statistical analysis

The incidence of ICU-acquired Gram-negative infections was described as a percentage. Patients who presented with an ICU-acquired Gram-negative infection (the study group) were retrospectively compared to remaining patients (the control group). The normal distribution of variables was evaluated using the Kolgorov-Smirnof test, and the results of continuous variables were presented as a mean ± standard deviation (SD). Categorical variables were analysed using the chi-square test and presented as a percentage.

Logistic regression analysis was performed to identify characteristics that, ceteris paribus, were associated with ICU-acquired Gram-negative infections and their relative weighting. In particular, a forward analysis was conducted in the observed population, starting from a null model and adding variables for the multivariate analysis. Results of multivariate analysis were presented as a p-value and odds ratio (OR) with 95 % confidence interval (95 % CI). Data were analysed using the STATA 9.1 software (STATA Corp, 4905, Lakeway Drive College Station, 77845, Texas, US).

## Results

Of the 494 patients enrolled in our study, 46 had an ICU-acquired infection (9.3 % of patients). In 30 out of these 46 infections, the isolated pathogen responsible was a Gram-negative bacterial strain (65.2 % of ICU-acquired infections). In the other 16 cases, a Gram-positive strain or a fungus were isolated.

**Fig. 1:**
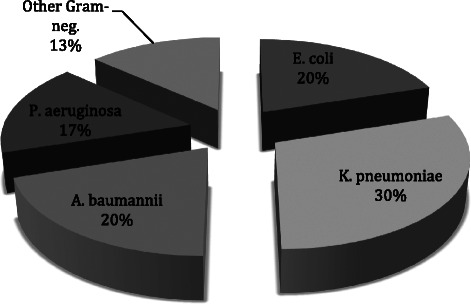
Primary ICU-acquired Gram-negative infections

**Fig. 2:**
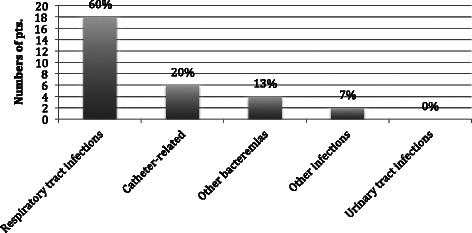
Sites of primary ICU-acquired Gram-negative infections

The Gram-negative bacteria responsible for the primary infections were: *K. pneumoniae* (30 % of ICU-acquired Gram-negative infections), *A. baumannii* (20 %), *E. coli* (20 %), *P. aeruginosa* (17 %) and in 13 % of cases the infections were caused by other Gram-negative bacteria such as *S. maltophilia, K. oxytoca, H. influenzae* and *Enterobacter spp* (Fig. 1)*.* The infection sites were mostly the respiratory tract (60 %), followed by catheter-related bacteraemia (20 %), non-catheter-related bacteraemia (13 %) and cutaneous (including surgical wound) infections (7 %) (Fig. 2).

Secondary infections were most commonly caused by *K. pneumoniae* (31.8 %), followed by *P. aeruginosa* (27.2 %), *A. baumannii* (22.8 %) and *E. coli* (18.2 %), and found in the respiratory tract (38.7 %), the bloodstream (25.8 %), peritoneal fluid (19.3 %), surgical wound infections (12.9 %) and the urinary tract (in only one patient).

Regarding antibiotics resistance rates, 90.4 % of isolated strains of *A. baumannii* were multi-drug resistant, and colistin was the only therapeutic option, while 81.8 % of isolated strains of *K. pneumoniae* were KPC-producers.

Amongst patients who developed one or more ICU-acquired infections due to Gram-negative bacteria, 40 % had been admitted for non-surgical treatment vs 8.1 % in the control group (*p* < 0.001); 23.4 % suffered from chronic renal failure vs 9.3 % in the control group (*p* = 0.018); and 33.4 % had been treated with antifungal agents before acquiring the Gram-negative ICU-acquired infections vs 10.3 % in the control group (*p* < 0.001) (Table 1).

**Table 1:** Characteristics of the population

	Total population	Gram-negative group	Control group	*p*
Age	69.6 ± 13.9	66.9 ± 10.7	69.8 ± 14.0	0.273
Male sex	63.1 %	76.7 %	62.3 %	0.120
BMI	26.1 ± 5.6	26.5 ± 5.5	26.1 ± 5.6	0.675
Medical admission	10.1 %	40.0 %	8.1 %	0.000
Hypertension	58.1 %	66.7 %	57.5 %	0.329
Valvular disease	5.9 %	10.0 %	5.6 %	0.328
Coronary artery disease	21.0 %	26.7 %	19.4 %	0.438
Heart failure	10.1 %	10.0 %	10.1 %	0.982
COPD	24.7 %	33.4 %	24.1 %	0.261
Diabetes	22.7 %	33.4 %	22.0 %	0.155
Chronic kidney disease	10.1 %	23.4 %	9.3 %	0.018
Malignancy	58.5 %	43.4 %	59.5 %	0.086
Previous antifungal therapy	11.7 %	33.4 %	10.3 %	0.000
Infection(s) on admission	9.5 %	20.0 %	8.8 %	0.051

Amongst patients admitted for surgical treatment, only 4.05 % (18/444) developed Gram-negative infections, while 95.95 % did not. Conversely, 24.0 % (12/50) of non-surgical patients developed Gram-negative infections, while 76.0 % did not.

The multivariate analysis showed that medical admission to the ICU and previous antifungal therapy were independent factors associated with Gram-negative ICU-acquired infections (*p* < 0.001, OR 7.12, 95 % CI 3.12 – 16.25 and *p* = 0.001, OR 4.05, 95 % CI 1.71 – 9.58 respectively) (Table 2). The average starting time from ICU-admission and the duration of antifungal therapy administration (expressed as days) between the Gram-negative group and the control group were 1.5 ± 0.3 vs 1.4 ± 0.4 (*p* = 0.179) and 7.1 ± 1.2 vs 6.8 ± 1.4 (*p* = 0.252), respectively.

**Table 2:** Multivariate analysis

	Odds ratio	*P* value	95 % conf. interval
Medical admission	7.12	0.000	3.12–16.25
Previous antifungal therapy	4.05	0.001	1.71–9.58

The comparison of ICU-length of stay (10.26 ± 2.43 vs 4.42 ± 2.37) and duration of mechanical ventilation (6.83 ± 1.53 vs 1.80 ± 2.42) showed that these were higher in the case group than in the control group (*p* < 0.001). However, no significant difference in mortality rate was observed between the two groups (16.67 % in the case group vs 12.39 % in the control group) (Table 3).

**Table 3:** Data on ICU outcome

	Total population	Gram-negative group	Control group	*p*
Days of mechanical ventilation	2.11 ± 2.7	6.83 ± 1.53	1.80 ± 2.42	<0.001
ICU length-of-stay	4.78 ± 2.75	10.26 ± 2.43	4.42 ± 2.37	<0.001
ICU- death	12.65 %	16.67 %	12.39 %	NS

## Discussion

The results of this study support the notion that the rate of new ICU-acquired Gram-negative infections is still high amongst a cohort of mixed medical-surgical patients, and mostly due to MDR bacteria. The relevant clinical factors independently associated with these infections were admission to ICU for medical reasons, chronic renal failure and fungal prophylaxis prior to bacterial infection.

More than 20 % of nosocomial infections are acquired in ICUs, and sepsis is a leading cause of death among patients, particularly those admitted to general and surgical ICUs [13]. In the Extended Prevalence of Infection in Intensive Care (EPIC II) study, the point prevalence of sepsis in ICU was 51 % in a cohort of 13,796 adult patients from 75 countries. Interestingly, 71 % of patients were receiving antibiotics on the day of study [1]. Infection in ICU was an independent predictor of mortality, with an estimated OR of 1.51 (*p* < .001) [1]. Data on the rate of new ICU-acquired infections are more difficult to obtain, partially because of non-homogeneous diagnostic criteria and different case-mixes. In a seven-year-long survey conducted by the National Nosocomial Infection Surveillance System on almost 500,000 patients admitted to 205 combined medical-surgical ICUs, the rate of ICU-acquired infection was 6.1 %, with surgical patients accounting for 50 % of these [14]. Recently, a French retrospective survey on patients over 80 years old showed a rate of ICU-acquired infection of 16.5 %, versus a rate of 13.9 % for younger patients [15]. In Italy, the 2006 surveillance program on infection in ICUs reported an ICU-acquired infection rate of 9.1 %, with MDR organisms accounting for more than 50 % of isolates and an associated ICU mortality rate close to 30 % [16]. In Spain, a rate of 9.3 % was recently found using data taken from 20 ICUs [17]. In our cohort, the rate of new ICU-acquired infections was 9.3 %, which was very close to that reported by Malacarne and colleagues. [16]. The difference in infection rates could be at least partially explained by the numbers of available beds in different countries. France, for instance, has an average of 38.5 ICU beds per 100,000 people [13], which may expose patients to the use of invasive devices that is invariably associated with ICU-infections.

In our cohort, 65.2 % of ICU-acquired infections were due to Gram-negative bacteria, with *K. pmeumoniae*, *A. baumannii*, *E. coli* and *P. aeruginosa* accounting for the vast majority of these (see Fig. 1). This finding was the same for both primary and secondary ICU-acquired infections. The most frequent types of infections were respiratory, catheter-related bacteraemia, non-catheter related bacteraemia, secondary peritonitis, surgical wound infections and a few urinary tract infections (see Fig. 2). Notably, 90.4 % of *A. baumannii* and 81.8 % of *K. pneumoniae* isolated strains were MDR. These figures are largely in line with data contained in current literature [18]. There is a wide consensus about the role of Gram-negative bacteria in causing most ICU-acquired infections. In the EPIC II study, the reported rate of Gram-negative isolates among ICU patient with infections was 62 %, with resistant *Staphylococci, Acinetobacter, Pseudomonas spp.* and *Candida spp.* accounting for the majority of these [1]. The high rate of Gram-negative bacteria isolated from patients with ICU-acquired infection has a twofold clinical significance: a high prevalence of MDR strains combined with limited therapeutic options; and a higher associated mortality, particularly for Gram-negative bacteraemia [13].

In the past years, Gram-positive bacteria, especially *S. aureus* and *Enterococcus spp*., developed a worryingly high rate of resistant strains. This led to intense efforts being made to discover new antibiotics that are effective against these microorganisms. New molecules active against VRSA and VRE, such as telavancin, are now available, and infections due to these pathogens, even when severe, can still be treated [19]. On the contrary, little or no progress has been made recently in the treatment of multi-drug resistant Gram-negative infections, even though they are currently a serious threat in many ICUs [20]. The lack of new antibiotics has led to older antimicrobial agents, such as colistin—formerly abandoned due to its toxicity profile—being considered. In many settings, this molecule is the only antibiotic effective on *A. baumannii* or *K. pneumoniae* [21, 22].

Regarding the associated factors, we observed that medical patients admitted to ICU were infected by Gram-negative bacteria more frequently than surgical patients. Actually, while 24.0 % (12/50) of medical patients developed Gram-negative infections, only 4.05 % (18/444) of surgical patients did so. Considering that most of the surgical patients did not develop a Gram-negative infection, this variable could not be considered as “risk factor” for this specific outcome, even if the absolute number of surgical cases with infection was higher than medical (18 vs. 12), due to the mostly surgical composition of the cohort. These results may be explained by the different clinical conditions for each group. Surgical patients were usually admitted to the ICU for post-operative management or, in the worst cases, for post-surgical complications, while medical patients were more often compromised, with many comorbidities, requiring more intensive and invasive treatments. Chronic kidney disease (CKD) was a factor associated with ICU-acquired Gram-negative infections, although findings in the literature mostly concern Gram-positive infections [23, 24]. The univariate analysis showed a double risk of infection in patients suffering from this condition, but the multivariate analysis did not confirm CKD as a factor independently associated with Gram-negative infections. Other conditions, such as the underlying causes of CKD itself (i.e., diabetes and hypertension) or hemodialysis, could therefore play a role.

Interestingly, we found that patients who acquired Gram-negative infections in ICU had been exposed to antifungal prophylactic administration more frequently than those who did not, and this turned out to be an independently associated factor. However, we did not find any significant difference either in the average starting time or in the duration of antifungal therapy administration between the Gram-negative group and the control group (1.5 ± 0.3 vs 1.4 ± 0.4, *p* = 0.179 and 7.1 ± 1.2 vs 6.8 ± 1.4, *p* = 0.252 respectively). The association between prior antifungal exposure and infection by Gram-negative strains could be related to some forms of bacterial-fungal interaction. Several studies show a variety of interactions between bacterial species and *Candida albicans*, demonstrated both in vitro and in animal models, which are mediated by mechanisms that are still not fully understood [10]. For instance, farnesol, a sesquiterpene molecule secreted by *C. albicans*, has been found to cause numerous antagonistic interactions between the fungus and various bacterial species. This molecule inhibits the production of virulence factors and alters quorum sensing of *P. aeruginosa*; it inhibits the viability of *A. baumannii* in biofilms; and it can also raise the susceptibility of *E. coli* and *S. aureus* to common antibacterial agents [25]. Moreover, emerging studies are investigating the field of bacterial-fungal interactions as a possible source of new therapeutic agents. In a recent paper, King et al. [26] show how a natural fungal product, *aspergillomarasmine A*., can restore the sensitivity to *carbapenems* in resistant strains of various pathogens by inhibiting NDM-1 and VIM-2—two clinically relevant *metallobetalactamases*. The available studies are mostly conducted in vitro or in animal models, and very little is known about these interactions in the clinical setting. To our knowledge, this is the first study showing a potentially relevant clinical effect of the indirect manipulation of these interactions with antifungal treatment. Data on this issue are not homogeneous, and a few studies have found that *C. albicans* colonization can be an independent risk factor for *P. aeruginosa* VAP [27]. Treating patients colonized by *C. albicans* may hence reduce the risk of *P. aeruginosa* VAP [28]. Our results are in contrast with these findings. However, groups of patients with different case-mixes may explain this discrepancy. In our cohort, due to the high prevalence of abdominal surgical septic patients, antifungals were administered pre-emptively without clear evidence of fungal colonization. Indeed, colonization by *Candida spp.* is a common finding in the ICU, and the risk of an invasive fungal infection often leads to an aggressive therapeutic approach, particularly in post-surgical septic patients. We suggest that an ‘over-liberal’ use of antifungal agents, despite their potential benefit to patients at high risk of fungal-related mortality, may have altered the bacterial flora of certain patients, contributing to colonization and subsequent infection by Gram-negative strains. Although this finding needs to be confirmed by larger, prospective studies, the association appears to be significant, and may lead to the consideration of a more conservative approach when deciding on the administration of pre-emptive antifungal therapy in the ICU.

In this study, the outcomes of patients with a Gram-negative infection were worse than those of non-infected patients in terms of length of ICU stay and duration of mechanical ventilation. Worse outcomes for critically ill patients with sepsis are well established in the literature [29, 30]. The mortality attributable to ICU-acquired infections is under debate, since a variety of factors (which are not always easily identified) can be influential here. In terms of survival rates, the current study showed no significant differences between infected and non-infected patients, although this study may have been underpowered to detect such a difference. Furthermore, we considered ICU-length of stay and duration of mechanical ventilation as outcome factors—i.e. total duration of stay in the ICU and total days of ventilation. Since these two outcome variables are also potential iatrogenic risk factors for infections, this result could reflect either a real difference in outcome between the two groups or an association between prolonged ICU-length of stay, prolonged mechanical ventilation and ICU-acquired infections. The retrospective nature of the study makes this difficult to ascertain.

Our study has several limitations. It was an observational, retrospective study conducted on a small sample group and involving a monocentric survey. Larger prospective studies are required to confirm our results. The retrospective nature of the study made it too difficult to obtain precise data regarding the reciprocal timing of events such as the discontinuation of mechanical ventilation and the collection of cultural specimens. This potential inaccuracy led us to consider some potential iatrogenic risk factors for ICU-acquired infections as outcome data (ICU-length of stay and duration of mechanical ventilation). For similar reasons, we have not considered differences in the severity of conditions on admission to the ICU as a potential factor associated with infection.

## Conclusions

In our cohort of patients, infections due to Gram-negative bacteria were common (65.2 % of ICU-acquired infections), admission to the ICU for non-surgical treatment and anti-fungal prophylaxis were found to be independently associated with these infections, and no significant differences were found in ICU mortality rates between infected and non-infected patients.

## Abbreviations

ICU: Intensive Care Unit
COPD: Chronic obstructive pulmonary disease
LOS: Length of stay
XDR: Extended-drug resistant
MDR: Multi-drug resistant
KPC: Carbapenemase-producing *Klebsiella pneumoniae*
SD: Standard deviation
OR: Odds ratio
CI: Confidence interval
VRSA: Vancomycin-resistant *Staphylococcus aureus*
VRE: Vancomycin-resistant *Enterococcus spp.*
CKD: Chronic kidney disease
